# High-throughput analyses of *Phocaeicola vulgatus* reveal fitness determinants for gut colonization and during colitis

**DOI:** 10.1080/19490976.2026.2661410

**Published:** 2026-04-23

**Authors:** Nazik M. Elmekki, Michael J. Coyne, Katia Flores, Leonor García-Bayona, Laurie E. Comstock

**Affiliations:** a Duchossois Family Institute, University of Chicago, Chicago, IL, USA; b Department of Microbiology, University of Chicago, Chicago, IL, USA

**Keywords:** Bacteroidales, *Phocaeicola vulgatus*, RB-TnSeq, colitis, RNASeq

## Abstract

*Phocaeicola vulgatus* is one of the most ubiquitous and abundant bacterial members of the human gut microbiota; however, the genetic factors that are essential for its survival and persistence in the mammalian gut and in a colitic host have not been analyzed. Using both RB-TnSeq and transcriptomic analyses, we performed genome-wide, unbiased analyses to identify genes important for growth in rich medium, those contributing to gut colonization, and those important during dextran sulfate sodium (DSS)-induced colitis. RB-TnSeq analyses of *P. vulgatus* from the feces of monocolonized mice identified 1189 genes that contribute to fitness in the gut. Transcriptomic analysis showed that the alternate sigma/anti-sigma factor gene pair *ecfO-reo* and the *nigD* genes of its regulon are amongst the most highly expressed genes in the mono-colonized mouse gut. Analyses of genes important during DSS-induced colitis identified a distinct set of genes, many of which are involved in nutrient acquisition. We extensively studied several genes affecting fitness by deletion mutant analysis with subsequent phenotypic characterization and by mouse competitive colonization analyses. Finally, we identified a previously undescribed sigma/anti-sigma factor pair that is drastically upregulated during DSS-induced colitis, along with a co-transcribed *spy* chaperone gene, known to help protect bacteria against numerous stressors. Altogether, this study provides the first comprehensive genome-wide analysis of *P. vulgatus* from the mouse gut and of any gut Bacteroidales strain during colitis.

## Introduction

Inflammatory bowel diseases (IBD) have long been correlated with alterations in the composition of the human gut microbiota as are an increasing number of non-GI human diseases.^1-5^ Bacteroidales is the most abundant and stable order of bacteria in the healthy human gut, accounting for 30%–40% of the total bacteria.^6^ Within the Bacteroidales, *Phocaeicola vulgatus* is one of the most prevalent and abundant species,^7^^,^^8^ yet it remains understudied compared to other gut Bacteroidales species, such as *Bacteroides thetaiotaomicron* and *Bacteroides fragilis.*^9-11^

In addition to thriving in the healthy gut, numerous Bacteroidales species withstand the inflammatory environment of patients with ulcerative colitis, Crohn’s disease, and other intestinal maladies and retain high levels of colonization.^6^^,^^12^ The bacterial factors that allow these bacteria to persist in an inflamed gut have not been examined. Different *P. vulgatus* strains have been shown to either exacerbate or protect against colitis.^12-16^ Genomic screens have identified genes of various Bacteroidales species important during various stress conditions,^11^^,^^17-20^ but analyses of genes contributing to the fitness of *P. vulgatus* or other gut Bacteroidales species during colitis have not been studied at the genomic level. We seek to increase our basic understanding of the mechanisms contributing to the persistence of *P. vulgatus* during colitis, which may improve the design of therapeutics or probiotic delivery methods to ameliorate colitis.^21-23^

This study presents high-throughput, genome-level analyses of a *P. vulgatus* strain isolated from a healthy human. We combine randomly barcoded transposon insertion sequencing (RB-TnSeq)^24^ and RNASeq as unbiased approaches for genome-wide screens in gnotobiotic mice. These analyses identified genes important for growth in rich medium, genes that contribute to colonization fitness in gnotobiotic mouse, and those that affect fitness during DSS-induced colitis. We performed targeted mutational analyses combined with phenotypic analyses, and mouse competitive colonization experiments to confirm the contribution of genes found to be highly significant for increased or decreased fitness.

## Materials and methods

### Strains and standard growth conditions

All strains and primers used in this study are listed in Dataset 1. *E. coli* strain AMD776 (the RB-TnSeq bank for Bacteroidales) was constructed in the Deutschbauer Lab.^11^ *E. coli* AMD776 was grown in Luria-Bertani (LB) broth supplemented with 0.3 mM diaminopimelic acid (DAP) at 37 °C.

*P. vulgatus* strain CL10T00C06 (PvCL10) was isolated from a healthy human donor.^25^ PvCL10 was grown at 37 °C under anaerobic conditions in basal broth^26^ with 0.5% glucose, 0.05% K_2_PO4, 0.05% L-cysteine, 5 µg/L hemin, and 2.5 µg/L vitamin K or on BHIS plates with the same hemin and vitamin K concentrations added.^27^

For sialic acid growth analyses, strains were grown anaerobically on BHIS agar plates, then single colonies were used to start 5 mL cultures in pre-reduced basal broth. After growth to the mid-log phase (OD ~0.4), 1.5 mL of each culture was pelleted and washed once with M9 medium (without glucose). After M9 wash, the pellets were resuspended in 500 µL of M9 medium without glucose and used to inoculate 96-well plates containing the various M9 media (either standard 0.5% glucose, 0.05% glucose, 0.05% glucose + 0.3% *N*-acetyl neuraminic acid (Carbosynth Batch MA007461101), and 0.3% *N*-acetyl neuraminic acid). Growth (OD_600_) was then measured every 30 min (shaking for 10 s prior to reading) using a Biotek Epoch 2 plate reader with Gen5 software (Agilent, Santa Clara, CA).

### *P. vulgatus* CL10T00C06 RB-TnSeq library construction

*E. coli* AMD776 was first grown to expand the library. A frozen aliquot was added to 100 mL of LB culture containing 60 mM DAP and 50 µg/mL carbenicillin and grown to an OD_600_ of 1.0, at which time 24 mL of glycerol was added, and 1 mL aliquots were frozen at −80 °C.

For conjugal transfer of the barcoded TnSeq plasmids to PvCL10, 3.5 mL of an overnight PvCL10 culture was added to 60 mL supplemented basal medium and grown anaerobically until OD_600_ of approximately 0.3 was reached. A 1 mL aliquot of the expanded *E. coli* AMD776 library was thawed and added to 40  mL of LB + DAP, then grown aerobically. After 5 h of growth, twelve conjugal matings were initiated by mixing 5 mL of PvCL10 and 1 mL of *E. coli*. These 12 mixtures were centrifuged for 10 min at 4300 × g and then each resuspended in 100 µL supplemented basal media with 60 mM DAP and spotted onto a BHIS plate and incubated aerobically for 18 h.

After 18 h, all 12 mating spots were resuspended into 22 mL supplemented basal medium and spread onto large BHIS agar plates with 10 µg/ml erythromycin (for transposon selection) and 200 µg/ml gentamycin (to kill *E. coli*). Smaller aliquots of the co-cultured strains were also spread on standard-sized BHIS agar plates with erythromycin and gentamycin to calculate the size of the library. Library plates were incubated anaerobically for 28 h, then all colonies were scraped into supplemented basal broth with erythromycin by repeatedly pipetting the liquid medium onto the plates, scraping, and pipetting off the suspended colonies. Glycerol was then added to a final concentration of 20%, and 2 mL aliquots were frozen at −80 °C. The insertion mapping was performed directly on bacteria scraped from the plates and combined with those after a 2-h passage in broth.

### Insertion mapping of the RB-TnSeq library

Genomic DNA was isolated from a 1 mL aliquot of the library using the Qiagen DNeasy UltraClean Microbial Kit (Germantown, MD, USA). The genomic DNA was then quantified by Qubit (Life Technologies, Carlsbad, CA, USA), and 1 µg was sheared using a Covaris model S2 ultrasonicator (Woburn, MA, USA) to create fragments with an average length of 300 bp. Fragments were then double-sided size-selected using AMPure XP beads (Beckman Coulter Inc., Brea, CA, USA). The NEBNext DNA library prep kit (New England Biolabs, Ipswich, MA, USA) was used for end repair, A-tailing, and adapter ligation. A two-step PCR was used to amplify fragments containing the transposon-insertion region, and the primers are listed in Dataset 1. The library was then sequenced using a MiSeq with 2 × 150 bp paired-end reads and a NovaSeq with 2 × 150 bp paired-end reads (Illumina Inc., San Diego, CA, USA).

The reads were aligned with the PvCL10 genome (NCBI RefSeq accession NZ_CP096965), and the barcodes associated with the insertion sites included 768,859 uniquely barcoded mutants. Barcodes were filtered out if they were mapped to multiple unique insertion sites or if the insertion site did not match an identified “TA” insertion site in the PvCL10 genome.

### Identification of genes with zero or drastically reduced insertions

Each gene was analyzed for the number of insertions at each TA site. Genes with zero insertions (after excluding the first 5% and last 10% of the gene) were included in the 382 gene list of Dataset 2, tab 1. To eliminate repetitive sequences from the essential gene list, which were likely miscalled owing to the inability to distinguish these elements during mapping, we first compared the PvCL10 genome to itself using blastn. ^28^ Hits larger than 1,000 bp and >90% ID were retained. Using these thresholds, the blast results revealed 350 reciprocal pairs between 1035 and 6064 bp. These DNA segments were retrieved and clustered using CD-Hit-EST^29^ (v 4.8.1, command line switches -sc 1 -sf 1 -d 0 -c 0.9 -*n* 5 -A 0.9 -g 1 -M 0 -T 80). This clustering analysis reduced the number of pairs to 24 clusters (Dataset 2, tab 2). A cluster representative was chosen and used as a query against the NCBI core nucleotide (core_nt) database using megablast (optimize for highly similar sequences). The megablast returns were parsed to retain only sufficiently sized hits against subject accessions CP096965.1, CP096966.1, CP096967.1, & CP096968.1 (i.e., arising from PvCL10). The chromosomal regions were then inspected to identify what genes they encoded. Many of the gene products were obvious proteins involved in mobility (e.g. transposases, IS elements, etc.). The products of those genes that were annotated as hypothetical proteins in the clusters were compared to one another using Clustal Omega^30^ to ensure that they were truly the same and were part of the repetitive element. If the repetitive span included a partial gene, those genes were ignored. As a result of these analyses, 94 locus tags were removed from the 382 gene list, and 4 others were manually removed, resulting in 284 remaining genes with zero mapped internal insertions (Dataset 2, tab 3).

### RB-TnSeq library analysis after growth under various conditions

For analysis of insertion sites from the RB-TnSeq bank propagated under various conditions, DNA was extracted (using either the Qiagen DNeasy UltraClean Microbial kit if broth or the Qiagen QiaAmp PowerFecal Pro if mouse fecal samples), then the barcode region was PCR-amplified in each sample with primers containing laddered indices, allowing demultiplexing to differentiate samples following pooled short-read sequencing (Dataset 7, tab 2). Barcode region amplicons were pooled by combining 100 ng of each sample, then cleaned using NEB PCR Clean-up kit and submitted for short-read sequencing (Illumina NovaSeq X, 2 × 150 bp reads) by the University of Chicago Genomics Core Facility.

We used the RTISAn pipeline for quantification of insertions in the output library.^31^ This pipeline first normalizes the barcode counts at each TA insertion site to account for replication bias and then compares the insertion counts between timepoints when analyzing two normalized “TAtally” counts. The “TAtally_in” file is repeatedly resampled based on the “TAtally_out” to simulate random loss of barcodes over time. These comparisons were calculated for individual mice, and then the script “combinereplicates.R” was used to identify genes significantly contributing to fitness across all mice and rank their contributions to fitness. Two independent colitis RB-TnSeq experiments were performed, each with one cage of each sex, and data from all mice were combined to determine their contributions to fitness (Expt 1 *n*   =   5, Expt 2 *n*   =   6).

### Assignment of genes to COG pathway functions

The 2024 COG^30^ PSSM models were downloaded from NCBI as a pre-compiled reverse position-specific BLAST database and compared to the PvCL10 proteome using the standalone RPS-BLAST program (v. 2.13.0+^29^). The returns were parsed and retained if the bitscore of the hit equaled or exceeded the bitscore threshold of the model as enumerated in the bitscore-specific scoring file (v 3.21), retrieved from NCBI's conserved domain database (CDD) site.^32^ Proteins with an above-threshold return were further assigned to the appropriate COG group and categories based on the COG model(s) returned.

### Construction of mutants Δ*upxY*, Δ*nanH*, and Δ*cirZ*

To construct deletion mutants, ~1 kb regions upstream and downstream of the region to be deleted were PCR amplified using Phusion master mix (New England Biolabs, Ipswich, MA) with the primers listed in Dataset 1. These flanking pieces were subsequently cloned into the BamHI site of pLGB13^27^using NEB builder Hifi assembly master mix (NEB) and transformed into *E. coli* S17λpir. Transformants containing the correct flanking regions were confirmed by whole-plasmid sequencing (Plasmidsaurus, Louisville, KY) and then conjugally transferred into PvCL10. For conjugal transfer, 1.6 mL of early log PvCL10 culture was mixed with 200 µL early log culture of the *E. coli* S17λpir containing the construct. Co-cultures were pelleted by centrifugation, resuspended in 100 µL of basal broth, spotted onto BHIS agar, and incubated aerobically for 18 h. Mating spots were then spread onto BHIS plates with 10 µg/ml erythromycin and 200 µg/ml gentamycin and incubated anaerobically. Colonies arising on these plates were screened for the correct insert by PCR, and confirmed cointegrates were passaged in 5 mL basal broth for several hours to allow for the second crossover event, then spread on BHIS plates with 75 ng/ml anhydrous tetracycline (aTc) for counterselection. The resulting colonies were PCR-screened to identify those with the mutant genotype. The resulting deletion mutants were whole-genome sequenced (Plasmidsaurus) to confirm the deletion.

### Construction of His-tagged NigD_3293_ and antiserum generation

His-tagged NigD_3293_ was produced by first generating a PCR amplicon that lacked the *N*-terminal signal sequence using the primers listed in Dataset 1, which was cloned into the NdeI site of pET16b. This construct was transformed into *E. coli* BL21 (DE3), and expression was induced at OD_600_ 0.4 with 0.5 mM IPTG for 3 hours. Induced bacteria were lysed by sonication, insoluble material was removed, and His-NigD_3293_ was purified from the soluble fraction using the ProBond purification system (Life Technologies/Thermo Fisher Scientific, Waltham, MA), dialyzed against PBS, and sent to LAMPIRE Biological Laboratories (Pipersville, PA) for polyclonal antiserum generation in rabbits using their ExpressLine protocol.

### PS promoter inversion analyses

To analyze whether the PS promoter was contained in an invertible region, we first used a PCR strategy involving a single forward primer (F) and two reverse primers (R1 and R2), each corresponding to either an ON or an OFF orientation (primers listed in Dataset 1). PCR reactions were performed using Taq 2X Master Mix (New England Biolabs, Ipswich, MA, USA) with an annealing temperature of 56°C, and amplicons were visualized using a 1.5% agarose gel. For each sample, F + R1 and F + R2 PCR reactions were performed. The F + R1 primer pair is expected to amplify a 350 bp product if the promoter is in the ON orientation, and the F + R2 pair is expected to produce a 430 bp amplicon if the promoter is OFF.

To quantitatively assess the relative abundance of the orientation of the invertible promoter within a sample, we performed a different PCR strategy^33^ in which the same forward primer as above was used with a different reverse primer that binds downstream of the inverted repeats (Dataset 1). This PCR product was then digested with HpaII (NEB), which cuts asymmetrically within the invertible region so that the on fragment would produce 431 bp and 149 bp digestion products while the promoter off orientation would produce 313 bp and 267 bp cleavage products.

### Growth of the bank with BcpT and DSS in broth

For RB-TnSeq experiments of broth-grown bacteria with various additions, analyses were performed in triplicate using three aliquots of the library. The mixture was added to 20  ml basal broth and grown anaerobically at 37 °C until an OD_600_ of 0.6 was reached. A total of 500 µL of each culture was then transferred to 5 mL basal broth with either 2.5% DSS or 6 µg/mL BcpT. These cultures were grown anaerobically at 37 °C until OD_600_ of 0.8 was reached, at which time 1.8  mL was pelleted and flash frozen on dry ice. DNA was extracted from the pellets using the Qiagen DNeasy UltraClean Microbial kit, and the barcode region was amplified from each sample using primers with laddered indices for demultiplexing and sequenced in one lane of a NovaSeq X (Illumina) with 2 × 150 bp paired reads.

### Animal colonization experiments

All mouse experiments were approved by the Institutional Animal Care and Use Committee (IACUC), University of Chicago. The mice were bred at the Gnotobiotic Research Animal Facility (GRAF) at the University of Chicago. After inoculation, the mice were housed in a cage rack system to maintain gnotobiotic status. The mice included both female and male germ-free C57BL/6J mice and were 7–10 weeks old at the time of gavage. Statistical comparisons between groups were performed with Graphpad Prism v10.5.

#### Monocolonization with PvCL10 and Δ*reo*

For these experiments, male mice were gavaged with 200  µl WT PvCL10 or Δ*reo,* and after 7 d of colonization, feces were collected and flash frozen for RNASeq analyses. Some of these fecal samples were also analyzed for the presence of NigD_3293_ in the bacterial fraction of the feces or in the fecal supernatant.

#### RB-TnSeq bank analyses in mice

Each mouse was gavaged with 200 µL of the RB-Tnseq bank. After 7 d, the drinking water of some of the mice was replaced with 2.5% DSS for 5 d and the mice were monitored for any changes in health. Fecal pellets were collected prior to DSS addition (D7), when DSS was removed (D12), and at the end of the experiment (D14). gDNA was extracted from the fecal pellets using the Qiagen QiaAmp PowerFecal Pro kit.

#### Competitive colonization analyses

Competitive colonization assays between WT and mutants were performed by gavaging mice with an approximate 50/50 mix of the WT and mutant strains, calculated based on the OD_600_ measurements prior to gavage. Subsequently, DNA was extracted using the Qiagen DNeasy UltraClean Microbial Kit for precise initial ratio analysis by qPCR. Fecal pellets were collected throughout the experiment until its endpoint on D14, and DNA was extracted using the Qiagen QiaAmp PowerFecal Pro Kit. DNA concentrations were measured by Qubit, and 20 ng DNA was used as a template for qPCR analysis of relative abundance using PowerUp SYBR Green Master Mix (Applied Biosystems/Thermo Fisher Scientific, Waltham, MA). Primers binding to the deleted gene were used to measure WT DNA, and primers binding to *gyrB* (a single-copy core gene, M0N98_00754) were used to measure total PvCL10 bacterial DNA (primers in Supp. Table 8). Each primer pair was found to have 90%–110% efficiency (calculated as E  =  10^(-1/slope)–1) using 5-point standard curves, and standard curves were used to calculate the absolute quantity of WT PvCL10 or total DNA in the sample. qPCR was performed using QuantStudio 6 Pro (Thermo Fisher).

### Western immunoblot analysis

For western immunoblot analysis, fecal pellets were resuspended in PBS (1 mL per 100 mg feces) and extensively vortexed. The particulate matter was allowed to settle, and 150 µL of the upper layer was combined with 50 µL of 4X lithium dodecyl sulfate (LDS) sample buffer, boiled, and insoluble material was removed by centrifugation. For analysis of the liquid broth cultures, the cultures were centrifuged, the pellets were resuspended in 150 µL of sterile water, and then prepared the same as fecal pellets. A total of 20  µl was then loaded onto NuPAGE 4%–12% Bis-Tris polyacrylamide gels with MES running buffer (Life Technologies). The contents of the gels were then transferred to PVDF membranes (Life Technologies). We started with an antiserum to whole-cell PvCL10 that was previously generated.^34^ To create an antibody fraction specific to the molecule lost in PvCL10Δ*upxY* mutant, we performed an antibody adsorption^35^ to remove antibodies to surface molecules common to both the wild-type and *upxY* mutant, leaving only antibodies to surface molecules lost in the mutant. PVDF membranes were probed with the adsorbed serum at a concentration equivalent to 1:3000 of the original whole-cell antiserum. Alkaline phosphatase-labeled goat anti-rabbit IgG (Pierce) was used as the secondary antibody, and following washing with Tris-buffered saline with 0.1% Tween, the membranes were developed with 5-bromo-4-chloro-3-indolyl phosphate/nitro blue tetrazolium (KPL, Gaithersburg, MD).

For analyses of NigD_3293_ in feces, we resuspended the fecal pellets in 10 volumes w/v sterile PBS, allowed the fibrous material to settle for 3 min, collected the upper layer containing bacteria, and additionally centrifuged this upper layer to remove bacteria to collect a fecal supernatant fraction. The equivalent of 3  µl of fecal material was loaded into each well of the gel. The western immunoblot proceeded as described above and the membrane was probed with anti-His-NigD_3293_ antiserum.

### RNASeq analysis

Bacterial samples for RNA sequencing were grown in triplicate in appropriate media to an OD_600_ of ∼0.8, pelleted anaerobically, and flash-frozen for processing by the Duchossois Family Institute Microbiome Metagenomics Facility (DFI-MMF). Following nucleic acid recovery using a Maxwell RSC instrument (Promega Corporation, Madison, WI), DNAse treatment and elution, the samples were quantified using Qubit (Life Technologies/Thermo Fisher Scientific, Waltham, MA), and integrity was assessed using a TapeStation 4200 (Agilent Technologies, Inc., Santa Clara, CA). Ribosomal RNA was depleted from all the samples using the NEBNext rRNA depletion kit (New England Biolabs, Ipswich, MA). The samples were fragmented, and the libraries were prepared using the Ultra Directional RNA Library Prep Kit for Illumina (NEB). Normalized libraries of biological triplicates of all samples were sequenced on the NextSeq 1000 platform (Illumina, Inc., San Diego, CA) at 2 × 100 or 2 × 150 bp read lengths.

For RNA sequencing from mouse feces, fecal pellets were collected from each of at least three mice on the post-gavage day indicated in the text, immediately placed on dry ice, and held frozen at -80 °C until processed by the DFI-MMF. These samples were processed as described above, except that each sample was first subjected to bead-beater disruption using a TissueLyzer II bead mill (Qiagen, Germantown, MD), and RNA was recovered via an RNeasy PowerMicrobiome Kit (Qiagen). Fecal pellets collected during DSS colitis were subjected to a LiCl-purification step with 7.5 M LiCl   +   50 mM EDTA to prevent any interference from the DSS.^36^

The reads were trimmed of adapter sequences and parsed for quality using utilities included in the BBMap package of bioinformatics tools (v. 38.90^37^) and mapped to the *Phocaeicola vulgatus* CL10T00C06 genome using the Bowtie 2 short-read aligner (v. 2.4.5^38^). The Bowtie 2 output was converted to sorted and indexed BAM files using SAMtools (v. 1.11^38^) and further processed using BEDtools (v. 2.30.0^39^) to enumerate reads mapped to coding regions of the *Phocaeicola vulgatus* CL10T00C06 genome.

These results were evaluated for differential expression using both DESeq2 (v. 1.48.1^40^) and edgeR (v. 4.6.2^41^) under R (v. 4.5.0, https://www.R-project.org).

The fastq read files for all samples used in the RNASeq analysis were deposited in the NCBI sequence read archive under BioProject ID PRJNA1428212.

## Results

### Library characterization and putative essential gene analysis

**Figure 1.**
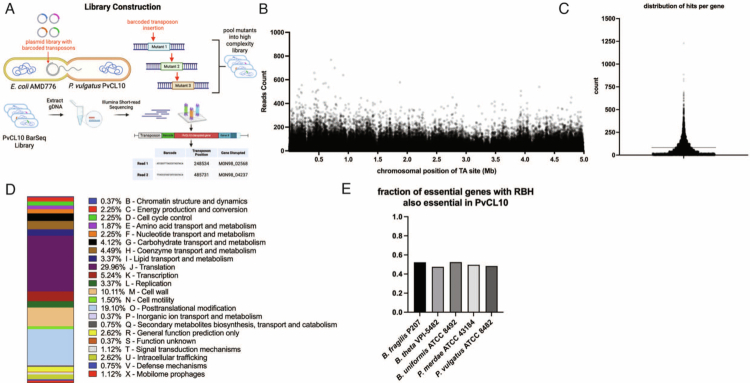
PvCL10 RB-TnSeq library characterization and essential gene analysis. (A) Schematic of library construction and insertion mapping process. (B) Number of insertion mapping reads at each TA site, plotted at PvCL10 chromosomal position. (C) Count of how many insertions were mapped per gene (mean 83.62, range 0–1227). (D) COG group assignments of protein-coding putative essential genes. (E) Fraction of essential genes of PvCL10 from other gut Bacteroidacae strains with a reciprocal best hit (RBH) in PvCL10 that were also essential in our PvCL10 analysis.

We constructed an RB-TnSeq library in *P. vulgatus* strain CL10T00C06 (PvCL10), a strain isolated from a healthy human,^25^ by conjugal transfer of the *E. coli* vector library AMD776^11^ (Figure 1A). The PvCL10 RB-TnSeq library comprises 768,859 barcoded mutants meeting our validation criteria (see Methods), with insertions distributed well across the PvCL10 chromosome, with a mean of 83.62 insertions per gene (range 0–1227 insertions) (Figure 1B, C). Essential gene analysis can be complicated by numerous factors, including insertions in genes that are not essential but that drastically decrease fitness, which will further decrease during library passage, insertions in repetitive sequences that cannot be properly mapped, insertions in non-essential domains of essential genes, and insertions in genes that are tolerated only when other genes are deleted, such as cognate immunity and toxin gene pairs. We began by taking a very stringent approach, compiling a list of genes with no mapped insertions outside of the first 5% and the last 10% of the genes, which is standard for these analyses.^42^^,^^43^ Of the 4319 annotated genes, 382 do not have mapped insertions meeting these criteria (Dataset 2, tab 1). Of these 382 genes, 98 are genes within insertion sequences or other sequences that are repeated numerous times in the genome (Dataset 2, tab 2). These genes could not be reliably mapped and, therefore, are not reported in the zero-insertion list. Among the remaining 284 genes (Dataset 2, tab 3) are those with essential functions, such as tRNA and rRNA genes, those encoding ribosomal proteins, DNA polymerase, and cell membrane assembly proteins. To analyze the broad functions of these 284 genes of PvCL10, we assigned COG groups^44^ to each gene product (Dataset 2, tab 4). Of the 4319 genes of the PvCL10 genome, 3913 (90.6%) genes were assigned to a COG group, with 181 of the 220 protein-coding genes of the 284 list assigned to at least one COG group. The largest COG group among these genes is “Translation” as expected (Figure 1D). To identify genes that are not on the 284 list but may be essential or confer severe growth defects, we analyzed all genes for the percentage of TA sites that have insertions and the average number of insertions per TA site (excluding the 5% and 10% of the termini of each gene). This analysis identified many genes with drastically reduced insertions or insertions at only one or a few sites, some of which may be essential genes such as arginine-tRNA ligase and DNA gyrase subunit A (Dataset 2, tab 4). We compared the 284 PvCL10 gene list to essential genes recently identified by RB-TnSeq analyses in five other gut Bacteroidales strains: *B. thetaiotaomicron* VPI-5482 (Bt-VPI),^11^ *B. fragilis* strain P207,^10^ *B. uniformis* ATCC 8492,^43^ *P. merdae* ATCC 43184,^43^ and *P. vulgatus* ATCC 8482.^43^ Using reciprocal best hit (RBH) analysis for protein-coding genes via blastp, we compared the fractions of essential genes in each of these strains to the 284 gene list of PvCL10 (Figure 1E). This comparison revealed that the 284 gene list lacks many of the essential genes common to all five other genomes (Dataset 2, tab 6). Our computations of the percentage of TA sites with insertions in each of these genes and the average number of insertions per TA site for each of the essential genes of other genomes absent in the 284 gene list (Dataset 3) revealed that many of the essential genes of the other species that are missing from the 284 gene list have drastically reduced insertions or insertions mapping largely to one or a few TA sites, typically in the 3’ end of the gene. Dataset 3 provides the insertion analyses of each TA site of each gene essential in the other species, yet absent from the PvCL10 284 gene list. Expanding the 284 list to include genes with insertions in 5% of internal TA sites (excluding the 5% and 10% at the termini of each gene) led to the inclusion of 57 genes that are considered essential in all five of the other Bacteroidales species but that were not present on the 284 gene list. This expanded list includes 193 of the 205 genes considered essential in all five other Bacteroidales strains (Dataset 2, tab 6).

### Genes contributing to fitness in the mouse gut

**Figure 2.**
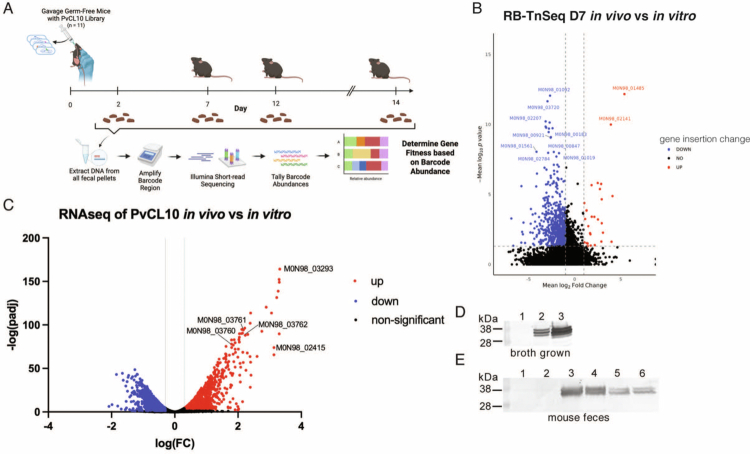
PvCL10 fitness determinants and differentially expressed genes in the gnotobiotic mouse gut. (A) Schematic of the RB-TnSeq experimental design in gnotobiotic mice. Analyses included in this figure were performed after colonization for 7 d (D7), but the mice were maintained for 14 d for DSS-colitis experiments outlined in Figure 3 (*n*   =   11, 2 independent experiments). (B) Volcano plot presenting the mean fold-change of normalized insertions in each gene comparing the barcode abundances from feces at D7 to the pre-gavage broth-grown culture. Designations of the most significantly different genes between the two conditions are listed. (C) Volcano plot of differentially expressed genes comparing bacteria from D7 mono-associated mice to broth-grown bacteria (*n*   =   3 per group). (D, E) Western immunoblots probed with antisera against NigD_3293_. (D) Lane 1 – Broth-grown cultures of PvCL10 cells and supernatant combined, lane 2 – cell lysate of Δ*reo*, lane 3 – supernatant of Δ*reo*. (E) Bacteria from the feces of pairs of mice colonized with Δ*ecfOΔreo* (lanes 1, 2) or with WT PvCL10 (lanes 3, 4). Lanes 5 and 6 were loaded with supernatant of feces of PvCL10 mono-associated mice. Each lane was loaded with the equivalent of 3 μl feces or an equivalent supernatant. The presence of multiple reactive bands is due to protein glycosylation, a property of most secreted proteins of gut Bacteroidales.^47^ NigD_3293_ has three glycosylation sites.

Compared to growth in rich broth, stable colonization of a gnotobiotic mouse gut requires many additional genes to obtain nutrients from both host and dietary sources, and to survive host antibacterial factors such as bile and antimicrobial peptides. Previous studies have examined genes contributing to the fitness of other gut Bacteroidales species in gnotobiotic mice,^17^^,^^18^^,^^45^^,^^46^ but to our knowledge, no such analyses of *P. vulgatus* have been reported. When *B. thetaiotaomicron* was introduced into gnotobiotic mice, genes reported to contribute to fitness included those encoding cell surface structures, vitamin B12 cofactors, and *rnf*-like oxidoreductase, among others.^45^ We gavaged germ-free mice with the PvCL10 RB-TnSeq library and analyzed the barcode abundances after 7 d compared to the T0 library sample pre-gavage (grown in broth) (Figure 2A). Based on our significance cut-offs (mean abs (FC)   >   2 and *p*-value   <   0.05 across all 11 mice), 1161 genes presented a significant decrease in insertions, suggesting that these functions increase fitness in the mouse gut, and 28 genes presented increased insertions (functions decreasing fitness under these conditions) (Figure 2B, Dataset 4).

**Figure 3.**
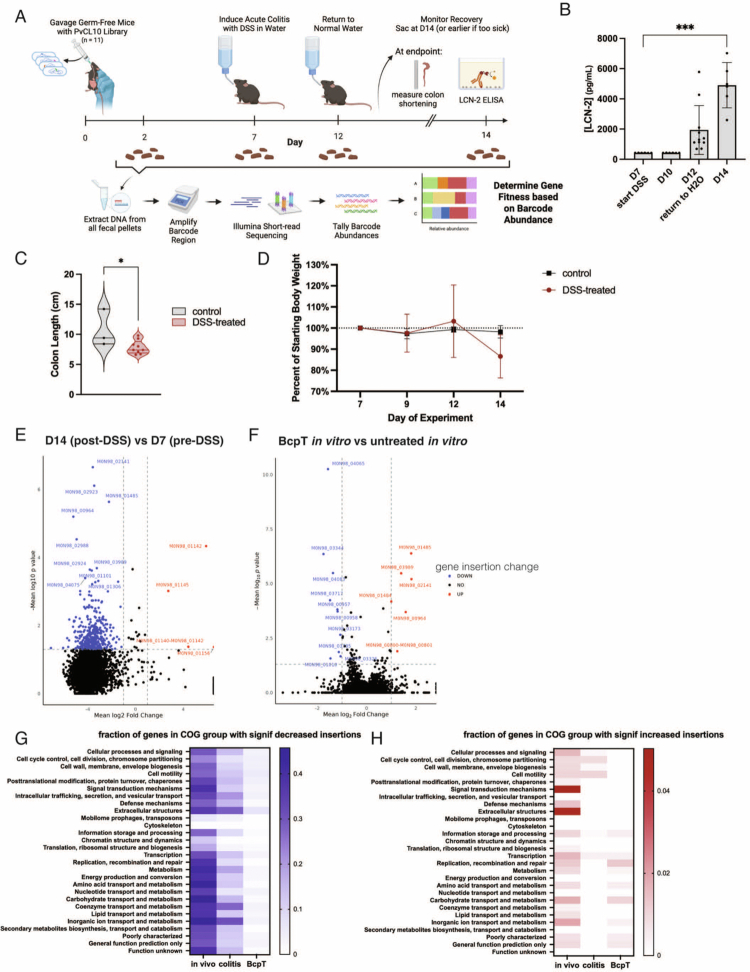
Fitness determinants during DSS colitis. (A) Experimental design for DSS-treated gnotobiotic mouse experiment (*n*   =   11, 2 independent experiments). (B) Fecal lipocalin-2 measured at various time points (D7 vs D14 *p* val 0.0007, Welch’s t-test). (C) Colon shortening measured on D14 at experiment endpoint (*n*   =   11 for DSS-treated, *n* = 3 for healthy controls; *p* val 0.0327, unpaired t-test). (D) Body weight loss of the mice measured during the DSS colitis experiment. (E, F) Volcano plots presenting the mean fold-change of normalized insertions in each gene. The designations of the most significantly different genes are listed. (E) D14 post-DSS compared to D7 pre-DSS. (F) Comparison of the effects of sublethal BcpT treatment in broth and pre-BcpT. (G) Fraction of total genes in each COG group with significantly fewer insertions from each experimental condition compared to pre-condition insertions. (H) Fraction of total genes in each COG group to pre-condition insertions.

We analyzed the COG assignments for these genes to understand which broad functions contribute to fitness. As not all PvCL10 genes are assigned a COG, while other genes are assigned to multiple categories, we report these genes as the fraction of total genes in a COG category that contributes to fitness under each selection condition. The three COG categories with the greatest fraction of *in vivo* fitness genes (genes with significant decreases in insertions) included amino acid transport and metabolism (category E), carbohydrate transport and metabolism (G), and inorganic ion transport and metabolism (P) (Figure 3G), indicating alterations in the acquisition of nutrients from the environment. The COG categories with the highest fraction of genes with significant increases in insertions included signal transduction mechanisms (T) and extracellular structures (W) (Figure 3H).

### Analysis of PvCL10 transcriptome in the mammalian gut

To obtain a more complete analysis of genes important for colonization, we performed an RNASeq analysis of bacteria from the feces of mice monocolonized with PvCL10 and compared the transcript levels of each gene to bacteria grown in rich broth. As expected, more than 43% of the genes were differentially expressed under these two conditions (Figure 2C, Dataset 5). Among the most upregulated genes in the mouse gut are a sigma factor‒antisigma factor pair (*ecfO-reo)* that we previously found to be the most upregulated genes when broth-grown bacteria are treated with either one of two different antibacterial toxins, BcpT or Bd-A.^48^ The antisigma factor Reo spans all five bacterial compartments, the cytoplasm, inner membrane, periplasm, outer membrane and outer surface, and likely senses outer membrane stress, resulting in the release of its cognate sigma factor, EcfO. In addition to *ecfO-reo*, three *nigD* paralogs that are part of the EcfO regulon^48^ are among the most highly upregulated genes in the mouse gut (Figure 2C). These genes are not only the genes most highly upregulated compared to broth-grown bacteria, but also among the genes most highly expressed in mono-colonized mice based on normalized TPM values (transcript per million) (Dataset 5, tab 2). The rank of genes based on *in vivo* expression, where the number 1 is the most highly transcribed gene *in vivo* (highest TPM) out of the 4138 protein-coding genes; three *nigD* genes were the 7^th^ (M0N98_03293), 18^th^ (M0N98_02415), and 29^th^ (M0N98_03762) most highly transcribed genes in the monocolonized mouse gut, which was even higher than most housekeeping protein-encoding genes involved in transcription and translation. Based on the high *in vivo* expression of *nigD* genes, we sought to analyze their production at the protein level. We generated a recombinant His-tagged NigD protein of M0N98_03293, the most highly transcribed *nigD* in the monocolonized mouse gut, and generated an antiserum to the recombinant protein. These analyses showed that this NigD ortholog is not produced in broth-grown cultures unless the antisigma factor is deleted (Δ*reo*), allowing the sigma factor EcfO to transcribe its operon (Figure 2D), or as previously shown, when the bacteria are exposed to antibacterial stressors, which induce the EcfO operon.^48^ The gut Bacteroidales genomes encode several NigD proteins, the functions of which are not described. One study using *B. thetaiotaomicron* showed that one of the NigD proteins of that species increases outer membrane vesiculation by an unknown mechanism.^49^ Using the antiserum to NigD_3293_, we show that in the Δ*reo* mutant, NigD_3293_ is not only cell associated, but also present in the supernatant, likely due to outer membrane vesiculation, as has been shown for other surface lipoproteins.^50-54^ In addition, we show that in the mouse gut, transcription of the three NigD proteins requires EcfO-Reo as there is no production of NigD_3293_ when mice are colonized with PvCL10 Δ*ecfOΔreo* (Figure 2E). These combined data confirm that NigD proteins are highly expressed *in vivo* and require EcfO for their transcription.

### Genes contributing to fitness during DSS-induced colitis

To investigate which genes contribute to fitness during colitis, we added 2.5% dextran sulfate sodium (DSS) to the drinking water of the mice after they were colonized with the PvCL10 RB-TnSeq library for 7 d (Figure 3A). Some of the mice were maintained with regular water for time-matched healthy control comparisons. After 5 d of DSS treatment (at D12), the mice were returned to normal drinking water for 2 d and sacrificed on D14. Fecal pellets were collected on D2, D7, D12, and D14. Fecal lipocalin-2 (LCN-2) was measured as a marker of inflammation and was found to peak on D14 (Figure 3B). Colon length was also shorter by a mean of 2.82  cm on D14 in DSS-treated mice compared to controls (Figure 3C). Body weight was measured throughout the experiment and was reduced to a mean of 86.6% of pre-DSS D7 body weight in DSS-treated mice compared to 98.2% in healthy controls on D14 (Figure 3D).

To identify genes contributing to fitness during DSS-induced colitis, normalized barcode counts were compared between the D14 (peak inflammation) and D7 (pre-DSS) timepoint within a mouse (raw reads plotted by TA site are shown in Figure S1B–E). Each gene’s contribution to fitness was ranked based on the average fold change and Mann‒Whitney *p*-value across all mice from two independent experiments. Comparing these two timepoints, 430 genes were significant during colitis compared to pre-DSS based on cutoff values of a mean fold change value ≥ 2 and a mean *p*-value of ≤ 0.05 across all mice (Figure 3E, Dataset 6). To determine if any insertions were significantly over- or under-represented due to the additional 7 d in the mouse rather than due to colitis, we compared the normalized barcode abundances of the healthy control groups of mice at D7 and D14. This analysis revealed 187 genes that met the significance cut-offs with 136 genes overlapping with those in the DSS colitis group (Fig S1F, Dataset 6, tab 2). As the fitness contribution of these genes is likely in response to increased colonization time in the mouse rather than to DSS treatment, we excluded these genes from the list of significant genes during DSS colitis. To further refine our list, we grew the RB-TnSeq library with 2.5% DSS in broth to identify genes that respond to DSS rather than induced inflammation (Figure S1G). This analysis revealed 19 genes with significantly decreased insertions when grown with DSS in broth culture; however, none of these overlapped with the DSS colitis fitness determinants (Dataset 6, tab 3). Altogether, these analyses identified 290 candidate genes with significantly decreased insertions due to DSS-induced colitis, and 4 genes with significantly increased insertions.

To determine if glycan/polysaccharide utilization is altered during colitis, we first identified the polysaccharide utilization loci (PUL) and glycoside hydrolases of the PvCL10 genome (Dataset 6, tab 1) using the CAZY PULDB.^55^ Of the 290 genes with decreased insertions during DSS colitis, 80 are within a PUL or encode a predicted glycoside hydrolase/polysaccharide lysase/carbohydrate esterase, showing extensive changes in nutrient acquisition during DSS-induced colitis.

Amongst the genes with significantly decreased insertions was *ecfO* (M0N98_03761), the gene described above that is substantially upregulated in the mouse gut (Dataset 5) and during treatment with the antibacterial toxin BcpT.^48^ These data suggest a broader stress‒response protective role for this sigma factor’s regulon. To determine if there are other overlapping genes important during colitis and exposure to BcpT, we treated the PvCL10 RB-TnSeq library with a sublethal concentration of BcpT in broth culture. Although this is a short-term exposure that does not allow for robust mutant selection, there were 61 genes with significantly fewer insertions and 6 genes with increased insertions (Figure 3F, Dataset 6, tab 4). COG analysis of the genes with significantly fewer insertions after BcpT treatment showed that the highest enriched fraction was in the extracellular structures category (Figure 3G). The genes with significantly fewer insertions that overlapped between the BcpT dataset and the DSS colitis dataset included a phosphofructokinase (M0N98_03989), an aspartate/alanine antiporter (M0N98_01484), and a cardiolipin synthase (M0N98_04083). Cardiolipin synthase has been shown in several bacterial species to be important during various stress conditions, including high salinity,^56^ desiccation,^57^ and recently shown to be important in *B. fragilis* during bile acid-induced stress.^58^ A phosphofructokinase was also reported as a fitness determinant during bile acid treatment.^10^ COG categories of colitis fitness genes revealed that the COG groups with the highest fraction of fitness conferring genes during DSS colitis are extracellular structures (W), coenzyme transport and metabolism (H), and inorganic ion transport and metabolism (*P*) (Figure 3G). Only four genes have significantly increased insertions during colitis, and all four belong to a single genetic locus that contains genes to produce a polysaccharide (PS) molecule (Figure 3E).

### PS decreases fitness in the DSS-treated mouse gut

Of the genes that significantly contribute to fitness during DSS colitis, two were selected for further investigation based on their predicted function and significance score: M0N98_01142, which encodes an UpxY transcriptional regulator, a family of regulators known to be essential for the production of numerous capsular polysaccharides of the gut Bacteroidales,^59^ and M0N98_03670, which encodes NanH, a neuraminidase that cleaves the terminal sialic acid from host glycans^60^ (analyzed in the next section). Mutants with insertions in these two genes did not significantly contribute to fitness between D14 & D7 in healthy untreated mice (*upxY*:healthy D14 mean *p*-value 0.48, ranked contribution to fitness 1444; *nanH:* healthy D14 mean *p*-value 0.105, rank 1115.5).

**Figure 4.**
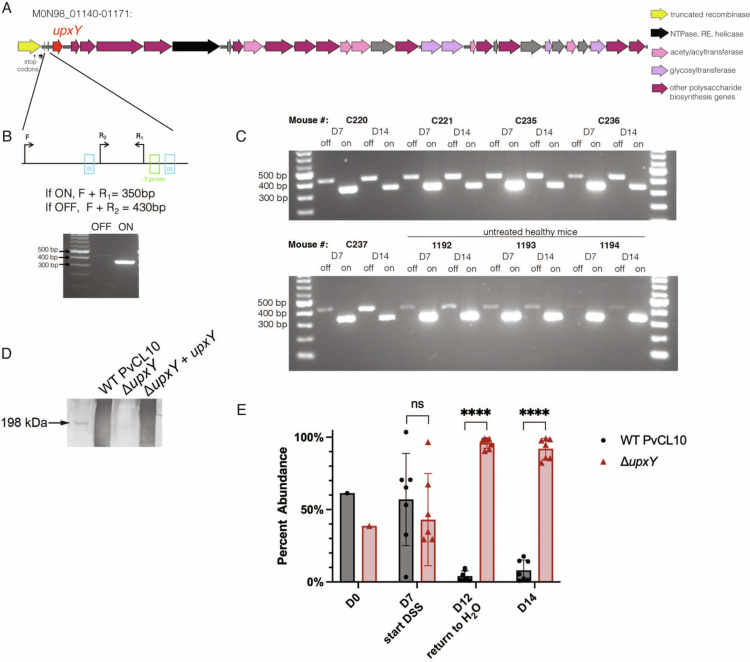
The PS locus with an increased number of insertions during DSS colitis encodes a large polysaccharide and contributes to fitness. (A) Map of the PS locus showing the *upxY* ortholog and other genes involved in polysaccharide biosynthesis. A gene with NTPase, restriction enzyme (RE), and helicase domains that interrupted the locus is shown in black. (B) Invertible promoter upstream of the *upxY* ortholog is shown in the promoter ON orientation. Inverted repeats are designated IRs. The -7 promoter region is highlighted in green. PCR design to determine if the region between the IRs inverts. The forward primer was used with each reverse primer. Below, agarose gel of the resulting PCR products from broth-grown bacteria. (C) Same PCR strategy as (B) but with gDNA extracted from mouse feces pre-DSS (D7) and post-DSS (D14) as well as from 3 healthy control mice that were not DSS-treated. (D) Western immunoblot of WT PvCL10, Δ*upxY*, and the complemented strain using adsorbed antisera showing the production of a high-MW, heterogeneously-sized polysaccharide that extends from less than 100  kDa to the top of the gel. (E) Competitive co-colonization experiment in gnotobiotic mice between WT PvCL10 and Δ*upxY*, relative abundances measured by qPCR (*n* = 7, D7 *p* val 0.7849, D12 & D14 *p* val < 0.0001, ANOVA with multiple comparisons).

M0N98_01142 (*upxY*) is the first gene of a predicted 30-gene PS biosynthesis locus (Figure 4A). There are increased insertions of this gene on D14 in the DSS-treated mice compared to the D7 pre-DSS mice, and there was no significant change between the healthy D7 mice and D14 mice, indicating that the effect was due to DSS-induced colitis. There were also increased insertions in three other regions of the same PS locus (M0N98_01145, M0N98_01156, and the intergenic region between M0N98_01140-M0N98_01142) after DSS treatment (Figure 3E). This finding suggests that the PS encoded by this locus decreases fitness during colitis.

Gut Bacteroidales polysaccharide biosynthesis loci are typically heterogeneous between strains of a species.^61-63^ Analysis of the genetic context of this locus and comparison with other *P. vulgatus* genomes revealed that this PS locus is located on a predicted 145-kb mobile genetic element (MGE). The presence of genes encoding type IV secretion system proteins involved in conjugal transfer suggests that the MGE is an integrative and conjugative element (ICE). The entirety of this PS locus is not present in other sequenced strains, although the locus was likely interrupted by the gene M0N98_01147 which encodes a large protein with an *N*-terminal P-loop NTPase domain, and a central helicase domain (Figure 4A).

Most capsular polysaccharides (CPS) and extracellular polysaccharides (EPS) of the gut Bacteroidaceae undergo phase variation due to an invertible promoter driving transcription of the respective locus.^33^^,^^64-66^ We identified a promoter flanked by inverted repeats upstream of *upxY* (Figure 4B). Site-specific recombinase genes are frequently upstream of invertible promoters in gut Bacteroidaceae species and mediate inversion of the downstream region. There is such a site-specific recombinase upstream of this PS invertible promoter; however, it is defective because numerous point mutations lead to internal stop codons truncating the protein. Using PCR analyses, we found that this promoter undergoes inversion (Figure 4B, C), which is likely mediated by another of the many site-specific recombinases in these bacteria. We detected the promoter on orientation almost exclusively in broth-grown bacteria (Figure 4B), but both orientations were detected in fecal pellets collected from healthy and DSS-treated mice (Figure 4C).

UpxY proteins are transcriptional antitermination factors that prevent premature termination of their respective locus.^59^^,^^67^^,^^68^ Thus, insertions in this gene should eliminate the production of the cognate PS. To test this prediction, we deleted M0N98_01142 (*upxY*) from WT PvCL10. We made an adsorbed antiserum to the ∆*upxY* mutant and used it for western immunoblot analysis. We found that *upxY* deletion abrogates the synthesis of a heterogeneously sized polysaccharide of very high molecular weight (>198  kDa) (Figure 4D). To validate that the production of UpxY decreases PvCL10 fitness during colitis, we performed an *in vivo* competitive colonization experiment between WT PvCL10 and ∆*upxY* in DSS-treated gnotobiotic mice using the same protocol as the RB-TnSeq experiment. We found that at D12 and D14, the ∆*upxY* strain almost entirely outcompeted the WT strain, supporting that the production of this PS reduces strain fitness during DSS-treatment (Figure 4E).

### Contribution of neuraminidase to fitness during DSS-induced colitis

**Figure 5.**
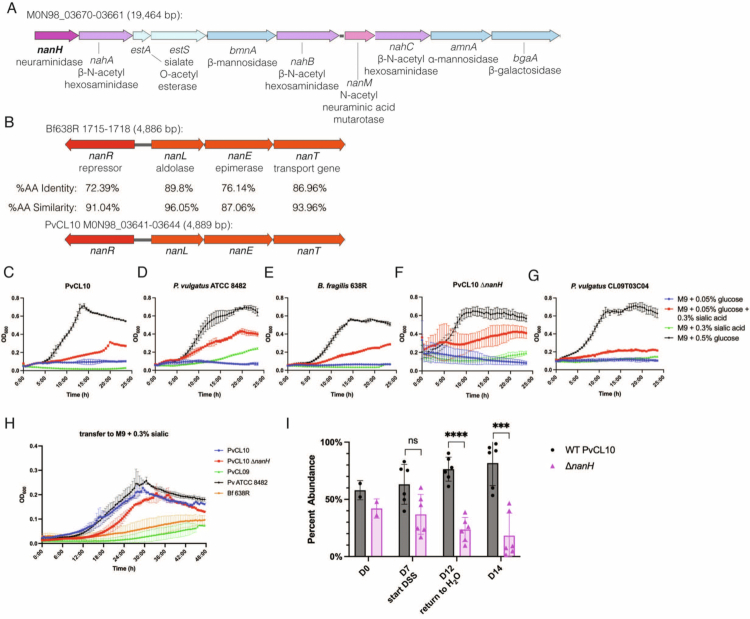
Neuraminidase provides a fitness advantage during DSS colitis. (A) Sialoglycoconjugate utilization (*sgu*) locus of PvCL10 containing *nanH*. (B) The second *nan* sialic acid utilization locus described in *B. fragilis* 638R has orthologs in PvCL10. The percent identity and similarity between proteins encoded by the two regions are shown. (C–G) Growth curves of PvCL10, Δ*nanH*, and other Bacteroidaceae strains previously shown to utilize sialic acid, each in triplicate. Defined medium with *N*-5-acetyl neuraminic acid alone (green), *N*-5-acetyl neuraminic acid and a low concentration (0.05%) of glucose (red), low glucose (0.05%) alone (blue), or normal glucose (0.5%) (black). (H) Transfer of 0.05% glucose   +   sialic acid cultures after growth to defined medium with sialic acid only. (I) Competitive co-colonization experiment of WT PvCL10 and Δ*nanH* in DSS-treated gnotobiotic mice (*n*   =   6, D7 *p* val 0.0764, D12 *p* val   <   0.0001, D14 *p* val 0.0007, ANOVA with multiple comparisons).

Unlike the PS, which decreased strain fitness during DSS-induced colitis, there were significantly fewer insertions in a neuraminidase gene (M0N98_03670, *nanH*) on D14 compared to D7 pre-colitis and D14 in healthy mice, suggesting that it is important for fitness during DSS-induced colitis (Figure 3E). *nanH* is the first gene of a 10-gene sialo-glycoconjugate utilization (*sgu*) locus previously described in *B. fragilis.*^60^ The genes of this locus are operonic and co-transcribed from a promoter upstream of *nanH* (Figure 5A). This *nanH-*encoded neuraminidase is expected to cleave sialic acid residues from host glycan molecules. Thus, the fitness advantage of the neuraminidase may be due to the removal of sialic acid, which would allow access to underlying sugars, and/or from the direct utilization of sialic acid. PvCL10 and other *P. vulgatus* strains have homologs of ten of the 13 genes of the *B. fragilis sgu* locus (Figure 5A). Another study in *B. fragilis* identified four additional *nan* genes (*nanR, L, E, T*) upstream of the *sgu* locus, which are required for sialic acid (*N*-acetyl neuraminic acid) utilization^69^ (Figure 5B). PvCL10 has homologs of all four of these genes (M0N98_03641-44), suggesting that it can utilize sialic acid as a nutrient.

Sialic acid utilization has been described as a property of some but not all *P. vulgatus* strains.^70^ To determine whether PvCL10 can utilize sialic acid, we grew it and three other Bacteroidaceae strains known to utilize sialic acid to varying degrees (*P. vulgatus* ATCC 8482, *P. vulgatus* CL09T03C04, and *B. fragilis* 638R) in minimal media with *N*-acetyl neuraminic acid, either as the sole carbon source, or with a low (0.05%) glucose concentration to initiate growth and allow transcriptional programming for sialic acid utilization (Figure 5C–G). We also grew the PvCL10 ∆*nanH* mutant under the same conditions to test whether deleting the neuraminidase would impact utilization, which it should not as this sugar does not need to be cleaved from a glycan chain under these growth conditions (Figure 5F). When each strain was added to defined M9 medium with sialic acid as the only sugar source, only Pv ATCC 8482 showed any level of growth (Figure 5D). However, all the strains, including PvCL10 and ∆*nanH*, grew when added to M9 medium with low (0.05%) glucose and sialic acid, albeit after a long lag phase (Figure 5C–G). We then transferred bacteria from these cultures to minimal media with sialic acid alone and observed that PvCL10 grew similarly to the known sialic acid utilizer, Pv ATCC 8482 (Figure 5H). This suggests that PvCL10 can directly use the sialic acid liberated by *nanH*.

To validate the fitness advantage conferred by *nanH*, we performed an *in vivo* competitive co-colonization experiment using DSS-treated gnotobiotic mice. WT PvCL10 outcompeted ∆*nanH* on D12, and ∆*nanH* continued to be slowly outcompeted at the terminal collection point at D14, supporting that this neuraminidase provides a fitness advantage during DSS-induced colitis (Figure 5I).

### Analysis of PvCL10 transcriptome during DSS colitis

**Figure 6.**
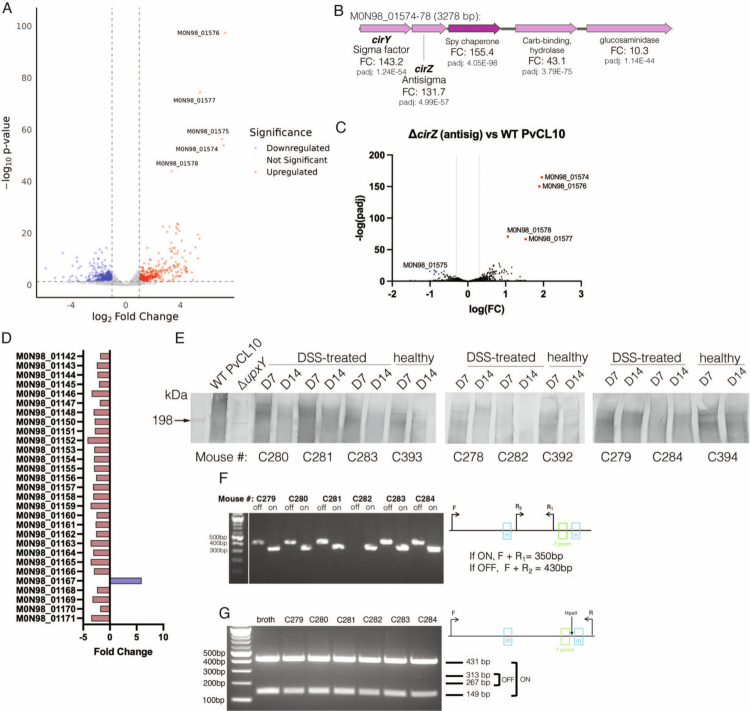
Transcriptomic analysis of PvCL10 during colitis and analyses of differentially regulated genes. (A) Volcano plot illustrating the differential expression of PvCL10 genes from mono-colonized mice on D14 after DSS treatment compared to untreated mice (D14) (*n*   =   7 DSS-treated, *n*   =   6 untreated). Five highly upregulated genes during colitis are listed. (B) Locus map of upregulated sigma/anti-sigma factor pairs and downstream genes with DESeq2 fold changes and *p*-values labeled. (C) Volcano plot showing the differential expression of genes of the PvCL10Δ*cirZ* mutant compared to WT PvCL10, both grown in broth. The regions shown in panel F are the most upregulated genes when the sigma factor gene *cirZ* is deleted. (D) Fold change in the expression of PS locus genes from DSS-treated mice compared to untreated mice (D14). (E) Western immunoblots of bacteria in fecal pellets from mono-associated mice probed with adsorbed antiserum to Δ*upxY.* The PS molecule is heterogeneously sized and appears as a high-molecular-weight smear, as apparent in the WT PvCL10 lane and absent in the ∆*upxY* lane. The mouse numbers are shown at the bottom. (F) (right) PCR strategy to determine whether the promoter upstream *upxY* of PS locus is present in both ON and OFF orientations from mono-associated mice. Agarose gel showing the results of the PCR from gDNA extracted from fecal pellets. (G) (right) PCR-digestion scheme to quantify the percentage of bacteria from mouse fecal samples with promoter in each orientation. (left) Agarose gel of PCR-digestion results with nearly all bacteria from mice with promoter in the ON orientation.

To expand our understanding of how PvCL10 responds to inflammation during DSS-induced colitis, we performed transcriptomic analysis of WT PvCL10 from D14 fecal pellets from mono-associated mice provided either normal drinking water or 2.5% DSS (D7–D12). Comparative analysis of these two conditions showed that 19% of the genes were differentially expressed, with 8% upregulated and 11% downregulated (Figure 6A, Dataset 7, tab 1). The most significantly upregulated genes during colitis compared to untreated mice are an ECF-type sigma/anti-sigma factor pair (M0N98_01574-75), upregulated 143- and 132-fold, respectively, and three adjacent genes (M0N98_01576-1578) whose expression was upregulated 155-, 43-, and 10-fold, respectively (Figure 6B). Neither this sigma/anti-sigma factor nor its regulon has been previously described; however, it is well-conserved across all 416 sequenced *P. vulgatus* strains analyzed. We designated these sigma/antisigma factor genes *cirY/cirZ* for colitis-induced regulators. Just downstream and co-transcribed with *cirYZ* is the most upregulated gene during colitis (M0N98_01576), which has strong structural similarity to the periplasmic chaperone, Spy.^71^ In other Gram-negative bacteria, Spy chaperones have been shown to protect against cell envelope stress by aiding protein folding in the periplasm and preventing protein aggregation.^72^^,^^73^ PvCL10 has another predicted Spy chaperone paralog (M0N98_01970) that is similarly downstream of a distinct sigma/anti-sigma factor pair and slightly upregulated during colitis (3-fold) (Fig S2A). Structural alignment of the Alphafold 3 predicted structures for these two Spy proteins suggests strong structural similarity, with a reported RMSD of 1.295 angstroms and the C-termini showing greater structural variation (Fig S2B).^74^ To elucidate the regulon of CirY, we deleted the antisigma factor gene *cirZ*, a mutant in which CirY is free to transcribe its regulon. RNASeq analysis of this antisigma factor deletion mutant showed that the genes most highly upregulated are *cirY*, *spy*, and the two downstream genes encoding a predicted carbohydrate binding hydrolase and a protein with glucosaminidase and LysM domains that may be involved in peptidoglycan binding and/or degradation (Figure 6B, 6C, Dataset 7, tab 2).

Despite the fact that *cirYZ*, *spy*, and the two downstream genes are upregulated during colitis, insertions in these genes in the RB-TnSeq bank are not underepresented during DSS-induced colitis (Dataset 6). Therefore, the environment in the mouse gut during DSS-induced colitis strongly induces this protective regulon, but it does not appear essential under these conditions.

We noted that all but one of the 30 genes of the PS locus described above were downregulated during colitis (Figure 6D). We used PS-specific adsorbed antisera to analyze fecal samples collected from the mice on D7 (pre-DSS) and D14 (post-DSS). The fecal samples from some of the mice showed reduced amounts of high-molecular-weight PS on D14, but this effect was not consistent across all mice (Figure 6E). We additionally investigated whether the invertible promoter upstream of *upxY* was flipped more toward the OFF orientation during colitis to explain the reduced expression. For this analysis, we performed the same PCR analysis of gDNA extracted from the fecal pellets and observed both ON and OFF amplicons from five of the six mice (Figure 6F). To quantify the fractions of the bacterial population that had the promoter in the ON and OFF orientations, we performed a quantitative PCR-digestion^33^ by amplifying the promoter region with primers that bind outside of the inverted repeat areas and then digested the PCR product with HpaII, which cleaves asymmetrically within the amplicon. By this quantitative method, we only observed bands corresponding to the promoter ON orientation, suggesting that the fraction of bacteria with the promoter off is extremely small. Therefore, other mechanisms may be responsible for reducing the expression of this locus during colitis (Figure 6G).

To determine if glycan/polysaccharide utilization loci are differentially expressed during colitis compared to the healthy gut, we identified which of the 56 PUL have genes with increased or decreased expression. Four PUL had at least one gene upregulated during DSS-induced colitis, including PUL44, which includes *nanH* identified in our RB-TnSeq screen^48^, and 24 PULs had at least one gene with decreased expression during DSS-induced colitis (Dataset 7, tab 1). Interestingly, PUL35, which comprises only a SusC/D pair (M0N98_01968-69), is immediately downstream of the second Spy chaperone region and is strongly downregulated during DSS-induced colitis (Fig S2A, Dataset 7). These data suggest that PvCL10 likely utilizes a subset of distinct nutrients during DSS-induced colitis compared to healthy conditions in the gut.

## Discussion

In genome-wide randomly-barcoded insertion sequencing analyses of bacterial strains, essential gene lists are typically provided. There are confounding factors to consider when compiling such lists, such as incorrect insertion mapping of repetitive genes, essential genes with non-essential domains that tolerate insertions, genes that are not essential but for which mutations are not tolerated unless adjacent genes are also mutated, polar effects in operons, and inclusion of genes that are not per se essential but that drastically reduce growth.^75^ This last factor varies depending on the number of passages prior to essential gene analysis. In this study, we do not provide an essential gene list but rather identify genes for which zero insertions were mapped (excluding the first 5% and last 10% of the gene) or for which insertions were tolerated at 5% or less of TA sites. The PvCL10 genome contains a substantial number of insertion sequences and repetitive elements (Dataset 2, tab 2) for which insertions cannot be correctly mapped. The removal of these genes resulted in 284 genes with zero insertions. As insertions are often tolerated in particular domains of essential genes,^43^^,^^76-78^ we also provide an analysis of the percentage of TA sites with insertions and the average number of insertions per TA site for each gene (Dataset 2, tab 5). Essential gene lists of five other Bacteroidales strains using RBH revealed 205 protein-coding essential genes common to all five genomes. The PvCL10 284 gene list contained only 136 of these genes. When genes with insertions in 5% of the TA sites of a gene were included in the list, this number expanded to 193 of the 205 genes essential in all five other strains (Dataset 2, tab 6). We provide in Dataset 3 an analysis of the number of insertions into each TA site of the essential genes shared in the five other genomes but missing in the 284 list, so that readers can make their own assessments of the essentiality of these genes and domains that may tolerate insertions. Even among the 284 genes in the list are those that are likely to tolerate deletion but for which the deletion would have a drastic growth defect. For example, *frdB* (M0N98_00548) encoding one of the fumarate reductase subunits, was on the 284 gene list and essential in the other five strains. We previously deleted this gene along with two adjacent genes encoding the other fumarate reductase subunits in *B. fragilis* and found that the strain has a drastic growth defect due to the loss of the ability to respire using fumarate as a terminal electron acceptor, but the strain is viable.^79^ However, deletion of this single gene may not be tolerated without deletion of the surrounding genes.

Unsurprisingly, colonization of the mouse gut requires substantially more genes compared to growth in rich broth. In fact, nearly 27% of PvCL10 genes have fewer insertions, many of which are involved in nutrient acquisition, including amino acids and glycans (Figure 3B, Dataset 4). It is expected that this gene list would vary compared to genes required to colonize a complex community. Such genes would be highly dependent on the community, as additional genes may be necessary under both exploitative and interference competitive interactions, whereas other genes may be conditionally dispensable due to nutrient cross-feeding.

RNASeq analysis of PvCL10 genes upregulated in the gnotobiotic mouse gut compared to broth-grown bacteria was very informative. We previously identified a protective sigma/antisigma factor pair of *P. vulgatus*, EcfO-Reo, that is drastically upregulated when PvCL10 is exposed to antibacterial toxins. EcfO-Reo were first characterized in *B. fragilis* and were shown to be induced after prolonged exposure to oxygen.^80^ The antisigma factor is unique because it spans all five compartments from the cytoplasm to the bacterial surface, and we propose that it responds to outer membrane stress. EcfO's regulon includes three genes encoding products of the NigD family of genes of unknown function. In this study, we show that *ecfO-reo* and the three *nigD* genes of its regulon are drastically upregulated in the mouse gut. In addition to being upregulated, TPM analysis revealed that these genes are among the genes most highly expressed in the gnotobiotic mouse gut (Figure 2C, Dataset 5). NigDs are of interest because they are ubiquitous in the gut of Bacteroidales species, each species encodes between 3 and 5 orthologs, they are upregulated during stress, and they are surface lipoproteins. In addition, one of the NigD proteins of *B. thetaiotaomicron* was shown to be involved in outer membrane vesicle formation.^49^ Using antiserum to NigD_3293_, we showed that this protein is not detectable in broth-grown bacteria, which is consistent with the lack of expression of *ecfO-reo* under non-stress conditions, but it is highly produced and secreted from bacteria when *reo* is deleted (Figure 2D). Bacteria from the feces of mono-associated mice showed strong NigD production that was detected not only in the bacterial fraction, but also in the fecal supernatant, showing it is secreted from the surface of the bacteria *in vivo* (Figure 2E). The same immunoblot analyses of mice mono-associated with Δ*ecfo-reo* showed that NigD production is completely dependent on this sigma-antisigma factor pair. Future studies should be aimed at determining the factor(s) in the gut that induce the EcfO/Reo stress response and the functions of the ubiquitous NigD proteins.

As the inflamed gut is a very different environment from the healthy gut, it is not surprising that there are 290 genes that are important for colonization and persistence during colitis. Though there is mixed literature on the impacts of *P. vulgatus* strains on IBD outcomes,^12-16^ our study design does not investigate the impacts of these genes on host disease. Some studies have reported increased *P. vulgatus* proteases in IBD cohorts compared to healthy,^12^^,^^15^ but we did not observe the impact of these genes on bacterial fitness. One of the most significant genes from our RB-TnSeq analysis is *nanH*, encoding a neuraminidase. The ability of PvCL10 to grow on sialic acid (Figure 4C) suggests that this fitness advantage may come from access to this nutrient during colitis or from access to the underlying sugars of mucin glycans that are accessible after sialic acid removal. It is possible that more host glycans are available to PvCL10 during DSS-induced colitis because of mucus sloughing. Though *nanH* is not upregulated during colitis, the *susCD* pair (M0N98_03671-72) directly upstream at the start of this PUL is upregulated (Dataset 7).

The only genes found to significantly decrease fitness during colitis are contained in a large PS biosynthesis locus. This locus is typical of CPS loci of the gut Bacteroidales in having an invertible promoter upstream of a gene encoding a UpxY family transcriptional antitermination factor. We analyzed the product of this locus, showing a high-molecular-weight PS that is produced in broth, in the mono-associated mouse gut, and during colitis, although its expression levels are decreased during colitis. The predicted DNA invertase upstream of the promoter is defective; therefore, our data show that this promoter, although able to invert minimally, is largely in the ON orientation under all conditions. The fitness disadvantage conferred by this locus during colitis may be because its synthesis is energetically unfavorable or because it may interact with the immune system in a manner unfavorable to bacterial persistence.

RNASeq analysis of PvCL10 from DSS-treated mice revealed a previously undescribed sigma/antisigma factor pair (*cirY/cirZ*) that is upregulated more than 130-fold during colitis (Figure 6A, 6B). The three genes downstream of *cirYZ* were also drastically upregulated. These three genes encode a*spy* ortholog, which is cotranscribed with *cirYZ,* and two downstream transcriptionally unlinked genes that may be involved in peptidoglycan binding and hydrolysis. Our follow-up transcriptomic analysis of Δ*cirZ* allowed the identification of the regulon of CirY showing that it most strongly upregulates transcription of itself, *spy* and the two downstream genes (Figure 6C). To our knowledge, the Spy chaperone has not been studied in the gut Bacteroidales. *In E. coli*, Spy is induced under cell envelope stress and binds to misfolded or unfolded substrates, allowing proper folding, and prevention of further aggregation.^72^ In *E. coli*, *spy* expression is controlled by the CpxAR and BaeSR two-component signal transduction systems,^81-83^ which respond to misfolded proteins in the cell envelope.^72^^,^^73^ This regulatory system is different in the gut Bacteroidales, where there are two distinct *spy* genes, both in operons with sigma/antisigma factors that are likely to respond to different envelope stresses. CirYZ and downstream *spy* are highly induced during DSS-induced colitis, whereas the sigma/antisigma factors upstream of the second *spy* paralog, although minimally upregulated during colitis, likely respond to other stress signals. The contribution of these regions to the persistence of gut Bacteroidales during gut stress certainly warrants further investigation.

Altogether, the high-throughput analyses of this study further provide important data concerning *P. vulgatus*, a highly prevalent member of the healthy human gut microbiome, and candidate species for targeted gut microbiome manipulation. It will be interesting to see how these data compare to those performed in other mouse colitis models. Further investigation of the genes and products identified in this study will increase our understanding of how a ubiquitous human gut symbiont responds to stress and could inform strategies for improved engraftment of *Phocaeicola vulgatus* as a therapeutic microbe.

## Supplementary Material

Dataset_4_RBTnSeq_in_vivo.xlsx Dataset_4_RBTnSeq_in_vivo.xlsx

Dataset_6_RBTnSeq_colitis.xlsx Dataset_6_RBTnSeq_colitis.xlsx

Dataset_7_RNASeq_colitis.xlsx Dataset_7_RNASeq_colitis.xlsx

Dataset_1_strains and primers.xlsx Dataset_1_strains and primers.xlsx

Dataset_3_reads_by_TA_position.xlsx Dataset_3_reads_by_TA_position.xlsx

Dataset_2_RBTnSeq_in_vitro.xlsx Dataset_2_RBTnSeq_in_vitro.xlsx

Dataset_5_RNASeq_in_vivo.xlsx Dataset_5_RNASeq_in_vivo.xlsx

Supplemental Figures.pdf Supplemental Figures.pdf

## Data Availability

The fastq read files for all samples used in RNASeq analysis were deposited in the NCBI Sequence Read Archive under BioProject ID PRJNA1428212.
