# Supplementary material for: High-throughput analyses of Phocaeicola vulgatus reveal fitness determinants for gut colonization and during colitis
Source: Gut Microbes. 2026 Apr 23;18(1):2661410. doi: 10.1080/19490976.2026.2661410 (PMC13114119; doi:10.1080/19490976.2026.2661410)
Supplement: Supplemental Figures.pdf [file KGMI_A_2661410_SM0280.pdf]

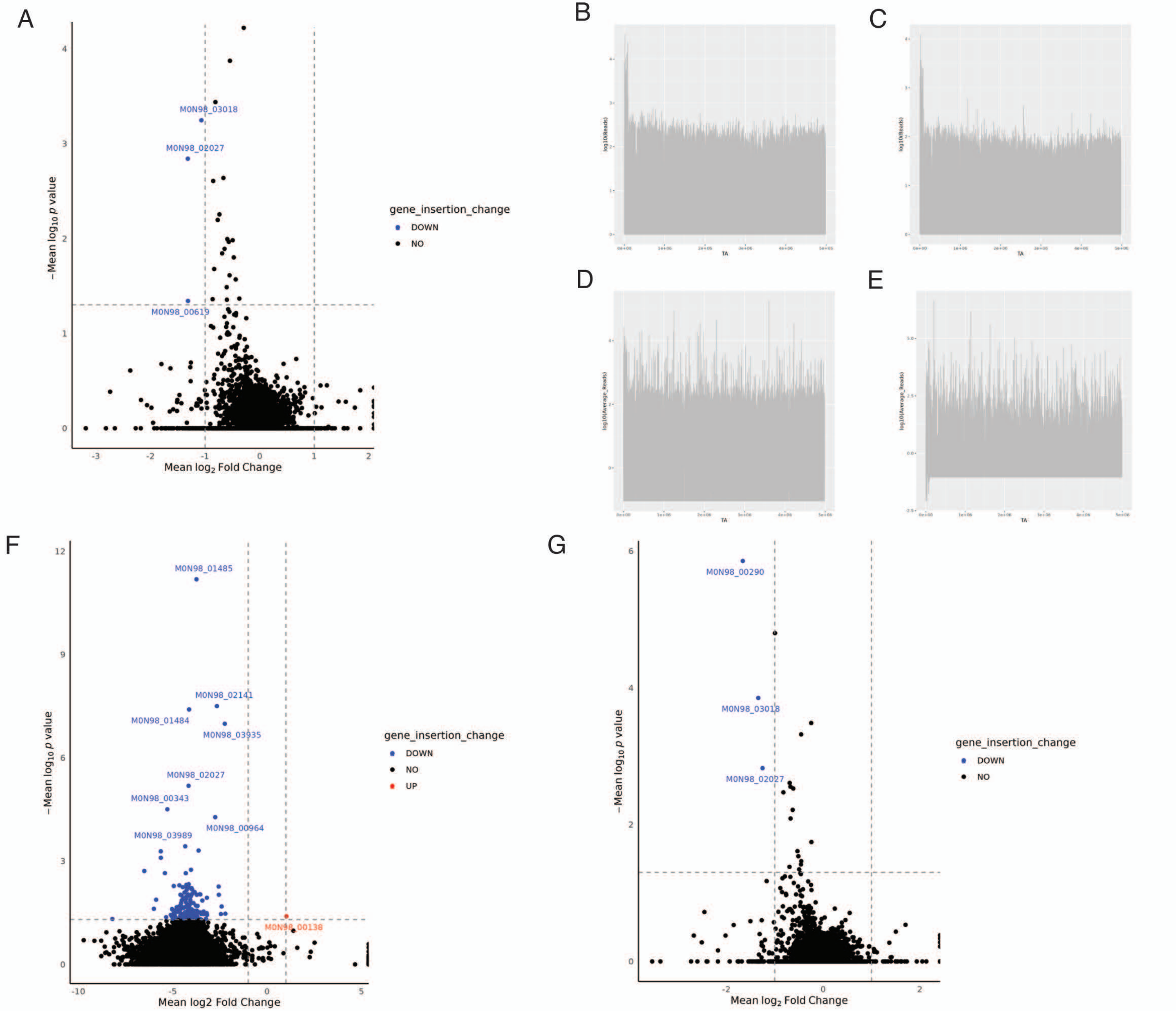


**Figure S1.** (A) Volcano plot of the untreated sample at OD 0.8 from *in vitro* RB-TnSeq experiment. (B-E) log transformed raw TA reads, (B) broth grown counts from insertion mapping. (C) DSS/*in vivo* T0 pre-gavage (D) D7 pre-DSS. (E) D14 post-DSS. (F) Volcano plot of RB-TnSeq data comparing healthy mice at D14 to healthy mice at D7. (G) Volcano plot of RB-TnSeq data of bacteria grown in broth 2.5% DSS compared to T0 broth pre-DSS.

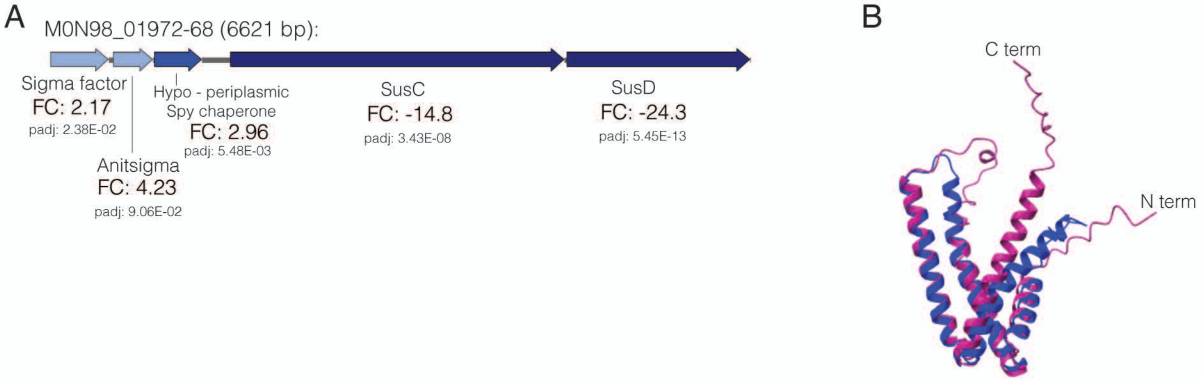


**Figure S2. Locus map of a second sigma/anti-sigma factor gene pair and co-transcribed spy upregulated during DSS-induce colitis, and a downstream *susCD* pair significantly downregulated during colitis.**
(A) Locus map of a second sigma/anti-sigma factor pair upregulated during DSS-induce colitis. Fold-change in gene expression of the locus during DSS-induced colitis compared to untreated mice are shown. A downstream susCD operon is significantly downregulated during DSS-induced colitis. (B) Alignment of the predicted structures of two the two putative Spy chaperones of PvCL10 using Alphafold (M1576 in magenta, 1970 in blue). Signal sequences were removed, pruned RMSD 1.295 angstroms.
